# Patient Eligibility and Results for Brain Metastasis in Phase 3 Trials of Advanced Breast Cancer: A Scoping Review

**DOI:** 10.3390/cancers13215306

**Published:** 2021-10-22

**Authors:** Renata Duchnowska, Everardo D. Saad, Małgorzata Banaszek, Ewa Pawłowska, Hanna Liberek, Natalia Cichowska-Cwalińska, Jacek Jassem

**Affiliations:** 1 Department of Oncology, Military Institute of Medicine, Szaserów St128, 04-141 Warsaw, Poland; 2 Dendrix Research, Sao Paulo 04508-011, Brazil; 3 Department of Oncology and Radiotherapy, Medical University of Gdańsk, 80-214 Gdańsk, Poland; mbanaszek@gumed.edu.pl (M.B.); ewanowak@gumed.edu.pl (E.P.); hania.lib@poczta.onet.pl (H.L.); nat.cichowska0@gumed.edu.pl (N.C.-C.); jjassem@gumed.edu.pl (J.J.)

**Keywords:** brain metastases, advanced breast cancer, randomized controlled trials

## Abstract

**Simple Summary:**

Even though up to 20% of patients with cancer eventually develop brain metastases (BM), most clinical trials have historically forbidden the enrolment of individuals with BM. The reasons for this practice include considerations regarding safety and efficacy, but there is a pressing need to verify whether new treatments also work for patients with BM. In this article, we assessed the literature on breast cancer and found that there has been an increase over time of trials allowing enrolment of breast cancer patients with BM, and that when the results for these patients were reported separately, they tended to go in the same direction as those observed for all patients. Our results suggest that further efforts are needed to increase the assessment of new treatments for patients with BM.

**Abstract:**

Background: Although brain metastases (BM) affect 5% of all breast cancer patients and 14% of those with metastatic disease, patients with BM are often excluded from participation in clinical trials. We conducted a structured assessment of the contemporary restrictions to enrolment of, and results for, patients with BM in phase 3 trials published over a period of 23 years in advanced breast cancer. Methods: We used PubMed to search for completed randomized trials published between 01/98 and 12/20. For all eligible trials, two authors independently abstracted data on general characteristics of the studies and detailed information on patient eligibility regarding the presence of BM. Results: We analyzed 210 trials, which enrolled 92,409 eligible patients. Of that total, 162 (77.1%) publications explicitly mentioned eligibility criteria related to the presence of BM and 75 (35.7%) trials reportedly allowed patients with BM, usually with restrictions related to prior brain treatment or stability of lesions. There was a significant increase over time in the percentages of trials allowing patients with BM (*p* < 0.001), and these trials were more frequently dedicated to HER2-positive or triple-negative disease (*p* = 0.001). Only 11 trials reported separate results for patients with BM at baseline. The direct treatment activity on BM was usually not reported, although in subgroup analyses the treatment effect in relative terms was usually better among patients with BM than in overall populations. Conclusion: Nearly 36% of phase 3 trials in advanced breast cancer over a 23-year period allowed patients with BM, and this practice is increasing over time. More research is needed to establish the activity of current and promising therapies in patients with BM.

## 1. Introduction

Although brain metastases (BM) affect up to 20% of individuals with cancer during the disease course [1], patients with brain or other central nervous system (CNS) metastases are often excluded from participation in clinical trials [2,3,4]. In some cases, exceptions are made to patients with treated, stable BM [5]. The main concerns regarding inclusion of patients with BM in clinical trials are usually their poor functional status, shorter life expectancy, increased risk of toxicity, and low expectation of treatment benefits (as a result of the blood–brain barrier) when compared with patients without BM; however, the frequency of BM in patients with cancer makes their systematic exclusion from clinical trials a potential source of bias in the assessment of novel treatments, thereby contributing to a plethora of reasons why trial populations are not fully representative of clinical practice [5,6,7]. Moreover, the fact that patients with BM tend to have a worse prognosis does not mean that they may not derive benefits from systemic therapy.

Breast cancer, which is currently the most frequent tumor type worldwide [8], is the second most common cause of BM, which have been estimated to affect 5% of all breast cancer patients and 14% of those with metastatic disease [9]. The incidence of BM in breast cancer appears to be rising, perhaps as a result of longer survival due to the improvement of systemic therapies that can control extracranial disease [10]. In breast cancer, the risk of BM varies according to phenotype and is reportedly higher in HER2-positive and in triple-negative rather than in luminal disease [10,11,12]. To our knowledge, no comprehensive assessment has been made of contemporary restrictions to enrolment of patients with BM in phase 3 trials in advanced breast cancer. In the current study, we present the results of such an assessment based on randomized trials published over a period of 23 years.

## 2. Materials and Methods

### 2.1. Identification of Trials

In order to identify eligible trials for analysis, we used PubMed and the medical subject headings ‘breast neoplasms’ and ‘drug therapy’, limiting the search to ‘randomized controlled trials’ published in the English language between 1 January 1998 and 31 December 2020. Eligibility required completed randomized trials to be explicitly qualified by their authors as phase 3 trials or to have at least 100 eligible patients per arm (unless explicitly qualified as randomized phase 2). We applied no restriction on the journal of publication but excluded articles reporting the results of companion studies on correlative biology or prognostic factors, trials on interventions other than systemic antineoplastic therapy, and trials using high-dose chemotherapy. For each eligible trial giving rise to more than one publication retrieved during the search, we analyzed the article considered to best represent the trial design and results. When the article identified in the eligible period was the long-term follow-up of a study originally published before 1998, we did not exclude the retrieved publication.

For trials published in the first two decades (i.e., before 2018), a second search was performed in an attempt to identify companion publications when an analyzed trial—retrieved by the above criteria—was found to allow inclusion of patients with BM. To this end, the names of the first and senior author of the analyzed publication were used alongside the name of one of the drugs used in the original article.

### 2.2. Data Collection and Statistical Analysis

Two authors independently abstracted data for all eligible trials and cross-checked each other’s work, including the exclusion of duplicates. When discrepancies were found, they were solved by the study supervisor (JJ). For each of the eligible articles, we abstracted data on the general characteristics of the trial, including the year and journal of publication, number of patients and comparison arms, primary endpoint, results on overall survival (OS) and progression-free survival (PFS), or time to tumor progression (TTP), when available, and detailed information on patient eligibility regarding the presence of BM. We made no distinction between different eligibility restrictions for parenchymal versus other types of involvement of the CNS, as most articles made no such distinction when describing eligibility criteria.

Given our chief goal of describing the practice in the literature regarding the questions of interest, we formally tested only a few hypotheses. In order to quantify the uncertainty around selected estimates, we computed 95% confidence intervals (CIs) for proportions. We compared categorical and numerical variables between groups of trials using Fisher’s exact test and the Mann–Whitney test, respectively, with a two-sided significance level of 5%.

## 3. Results

### 3.1. General Characteristics of the Trials

**Figure 1:**
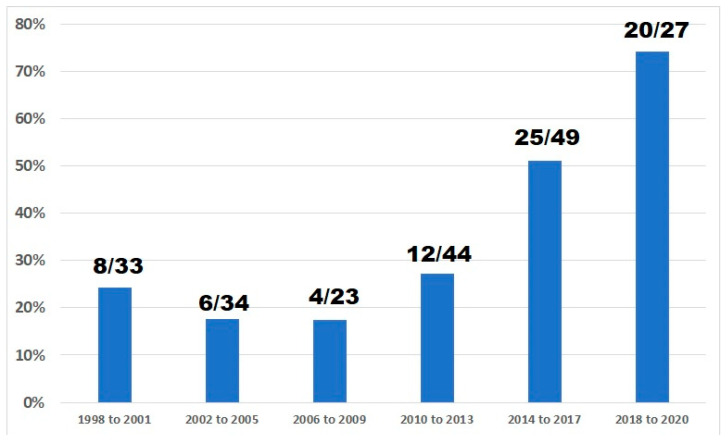
Percentages of trials allowing patients with brain metastases according to years of publication (fractions indicate number of trials allowing patients with brain metastases over all trials in the period).

We analyzed a total of 210 randomized trials, which enrolled a total of 92,409 eligible patients (Supplementary Table S1). Ninety (42.9%) trials were published between 1998 and 2009 and 120 (57.1%) were published between 2010 and 2020 (Figure 1). Most (189, 90.0%) of the trials had two arms, 20 had three arms, and one had four arms; the median number of patients per arm was 182 (range, 39 to 677). A total of 119 (56.7%) trials exclusively assessed patients in the first line of therapy, 50 trials contained patients in the first or subsequent lines, and 41 exclusively contained patients in subsequent lines. With regard to the breast cancer phenotype, 111 (52.9%) trials enrolled patients with various phenotypes, 40 (19.0%) were dedicated to hormone-receptor-positive disease alone, 33 (15.7%) to HER2-positive disease (regardless of hormone receptors), and seven trials (3.3%) to triple-negative breast cancer. For 19 trials, the eligibility based on phenotype was not reported (14 of these trials were published in the first 12 years of interest). Only six of 81 trials published in the first 12 years were dedicated to HER2-positive disease, compared with 27 among the 120 in the second 11 years. The primary endpoint was OS in 12 trials, PFS or TTP in 125 (59.5%), response rate in 35 (16.7%), quality of life in two, and other endpoints in 17 trials; 19 (9.0%) trials had co-primary endpoints.

### 3.2. Eligibility and Reporting of Patients with Brain Metastases

**Figure 2:**
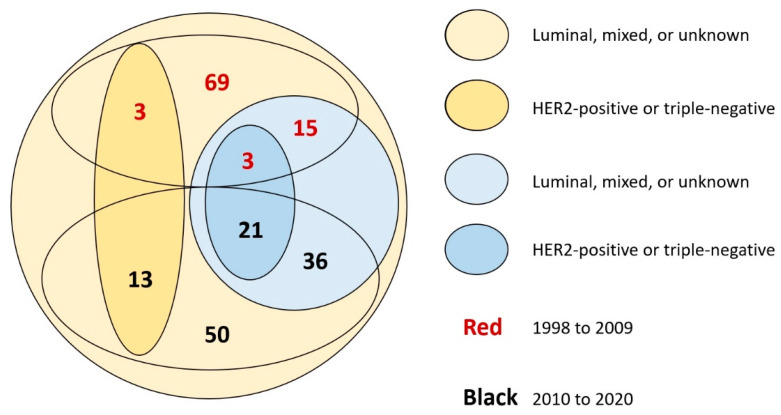
Numbers of trials according to decade of publication (red or black font, respectively, for first versus second decade), allowance for brain metastases (yellow or blue, respectively, for no versus yes), and phenotype (light or dark color, respectively for luminal, mixed, or unknown versus HER2-positive or triple-negative).

Among the 210 trials, 162 (77.1%) published results explicitly mentioning eligibility criteria related to the presence of BM, while in 48 articles (22.9%) there was no information on the eligibility of such patients. Of the 210 trials analyzed, 75 (35.7%; 95% CI, 29.2% to 42.6%) allowed patients with BM; these trials included a total of 31,650 patients (34.2% of the total). Only five of the 75 trials allowing patients with BM reportedly applied no restrictions, 66 applied restrictions (usually related to prior radiation or stability of current brain lesions), and in four cases there was no information. Figure 1 displays significantly increasing percentages of trials allowing patients with BM for each 4-year period (or for 3 years in the case of trials between 2018 and 2020; Chi-square for trend, *p* < 0.001). There were no significant differences in the median number of patients per arm or in the proportion of trials of first-line therapy exclusively between trials allowing and not allowing patients with BM (data not shown). On the other hand, 24 of 75 trials reportedly allowing patients with BM were dedicated to HER2-positive or triple-negative disease (vs. hormone-receptor-positive, mixed, or unknown), compared with 16 of 135 trials not allowing patients with BM (*p* < 0.001; see Figure 2).

### 3.3. Specific Results for Patients with Brain Metastases

Only six of the trials published before 2018 reported separate results for patients who had BM at baseline in the same article with the main results (Table 1) [13,14,15,16,17,18,19]. Moreover, 19 of the other 55 trials allowing inclusion of patients with BM had at least one companion publication; in one of these we could find subgroup analyses for patients with BM, totaling seven trials with such separate results (Table 1) [20,21]. All of those trials were published between 2008 and 2017. Finally, four trials published between 2018 and 2020 reported separate results for patients who had BM at baseline in the same article with the main results (Table 1) [22,23,24,25,26]. Three of the 11 trials enrolled patients in the first line, two in the first and subsequent lines, and six only enrolled patients in second or subsequent lines of therapy. Those 11 trials had a median of 301 patients per arm, compared with a median of 176 for the 199 trials not reporting separate results for patients with BM (*p* = 0.0131). Seven of the 11 trials reporting separate results for patients with BM were dedicated to HER2-positive disease, compared with 26 of 199 trials not reporting those separate results (*p* = 0.009).

The comparative efficacy results between the overall population and the subgroup of patients with BM for the 11 trials reporting such results separately are summarized in Table 1. The direct treatment activity on BM lesions present at baseline was reported in only one trial, in the form of PFS in the central nervous system. For the trials reporting subgroup analyses defined by the presence of BM, as a general rule the treatment effect—on PFS in four cases and OS in four—was similar or better among patients with BM at baseline when compared with the overall results for each trial. There were two trials in which a beneficial treatment effect on PFS was absent among patients with BM but present overall, and in one of these trials there was a treatment effect on OS in the subgroup with BM despite the absence of PFS benefits.

## 4. Discussion

Our results show that over a period of 23 years, nearly 35% of randomized trials in advanced breast cancer reportedly allowed patients with BM, and that these trials enrolled nearly 34% of the total number of patients represented in this literature during that period. As many as 48 articles did not mention the eligibility of patients with BM; however, we did not look at trial protocols, but rather relied on publications to ascertain eligibility with regard to BM. As a result of this practice, it is possible that these 48 trials for which the published articles did not explicitly report on the presence of restrictions to the enrolment of such patients or results for this subgroup did indeed have explicit criteria; therefore, we may have slightly underestimated or overestimated the true proportion of trials actually allowing enrolment of patients with BM. Nevertheless, our results show that the enrolment of patients with BM has nearly tripled over the period of interest. These findings are likely due, at least in part, to the increased proportion of trials dedicated to HER2-positive breast cancer, which were more likely to allow BM.

Similar observations in HER2-positive, advanced breast cancer come from the systematic review by Costa et al. of phase 1, phase 1/2, and phase 2 trials published between 1992 and 2016 [3]. These authors found that 29% of early-phase trials allowed the inclusion of patients with CNS metastases, a practice that they found to be more frequent among trials dedicated to HER2-positive disease and those on targeted therapies. As in the present study, the inclusion of patients with CNS metastases increased over time.

Using the ClinicalTrials.gov website, accessed on 7 August 2021, McCoach et al. performed a systematic search of eligibility regarding brain and leptomeningeal metastases in trials enrolling patients with advanced non-small-cell lung cancer [4]. This is of particular interest, given that lung cancer is the leading cause of BM [1,9]. They found that 14% of trials excluded patients with CNS metastases, 41% allowed enrolment after local treatment, and only 26% allowed patients with no prior treatment for CNS lesions; therefore, the total percentage of trials in advanced non-small-cell lung cancer reportedly allowing patients with BM (67%) appears to be twice as high as the corresponding percentage we found in breast cancer (35.7%). Interestingly, no explicit mention of CNS metastases was made in 19% of trials retrieved from ClinicalTrials.gov, accessed on 7 August 2021, a percentage remarkably similar to the one we found in published trials in advanced breast cancer (22.9%). In the multivariate analysis by McCoach et al., only the sponsor type was predictive of exclusion of patients with CNS metastases, with industry-sponsored trials being more likely than academic trials to exclude those patients. The authors concluded that direct evidence of activity of a treatment on CNS metastases cannot be reliably generated in most trials in non-small-cell lung cancer, encouraging sponsors to consider the exploration of benefits for patients with BM more explicitly [4].

BM remain a largely unmet need in advanced breast cancer, particularly in HER2-positive and triple-negative disease. Improved control of extracranial disease through the use of anti-HER2 agents may increase the problem, something that further highlights the need to assess novel agents for their activity in the brain [27]. Recently, the U.S. Food and Drug Administration (FDA) approved tucatinib in combination with trastuzumab and capecitabine for patients with advanced HER2-positive breast cancer, including patients with BM, who have received one or more prior anti-HER2-based regimens for metastatic disease; importantly, this was the first FDA approval that specified BM in the indication statement [24,28]. Interestingly, the addition of tucatinib to the backbone therapy led to a PFS treatment benefit that was of nominally higher relative magnitude than that observed in the overall population of the pivotal trial [23,24]. This observation is similar to findings reported from three of the other trials described in Table 1 [13,15,19,21]; therefore, it is vital to distinguish the prognostic from the predictive roles of BM. Even though the presence of BM usually confers a worse prognosis, the results in breast cancer suggest that patients with these lesions derive at least the same relative magnitude of benefit from treatment than patients without BM. Of note, the findings from one of the trials shown in Table 1 [17] led the authors to design a new phase 3 trial of the same agent exclusively among patients with BM [29]. Likewise, ongoing combination therapy trials for patients with BM from breast cancer have been recently summarized [30].

## 5. Conclusions

In conclusion, nearly 36% of phase 3 trials in advanced breast cancer reported over a period of 23 years have allowed patients with BM, and this practice has been increasing over time. Moreover, trials published within the last three years have allowed the enrolment of such patients in nearly 75% of cases. The proportion of trials in HER2-positive and triple-negative breast cancer has increased in parallel, probably explaining the trend regarding allowance for BM; however, there have been very few BM-specific trials, and efforts are needed to increase their number. One important caveat in that regard is the adequate use of patient selection and evaluation criteria, as well as of backbone therapies with proven activity against BM [28]. Also important is the increased reporting of results relating to the subgroup of patients with BM, a relatively infrequent practice in earlier studies.

## Figures and Tables

**Table 1:** Trials reporting separate results for patients with brain metastases at baseline.

Author (Year)	Line of Therapy	N Total	N with Brain Metastases	Phenotype	Treatment Effect in Overall Population and Subgroup with Brain Metastases
Blackwell (2010) [13]	Second and subsequent	296	36	HER2-positive	Direct efficacy on BM not reported. PFS was not significantly different between control and experimental arms among patients with BM, but the HR in these patients (~0.60) was lower than the overall HR (0.73).
Stockler (2011) [14]	First	323	150 *	Mixed	Direct efficacy on BM not reported. Comparison between overall population and subgroup not reported.
Verma (2012) [20] and Krop (2015) [21]	First and subsequent	991	95	HER2-positive	Of 95 patients with BM at baseline, 18 developed BM progression on study. There was no difference in PFS between control and experimental arms (HR = 1.00) among patients with BM at baseline, unlike the PFS superiority for experimental treatment overall (HR = 0.65). Overall survival was significantly lower in the control than in the experimental arm among patients with BM, with HR of 0.38 in these patients versus 0.68 overall.
Krop (2014) [15] and Krop (2017) [16]	Second and subsequent	602	72	HER2-positive	Direct efficacy on BM not reported. PFS was not significantly different between control and experimental arms among patients with BM, but the HR in these patients (0.62) was lower than the overall HR (0.68). Overall survival was significantly lower in the control than in the experimental arm among patients with BM, with HR of 0.62 in these patients versus 0.69 overall.
Perez (2015) [17]	Third and subsequent	840	67	Mixed	Direct efficacy on BM not reported. Overall survival was significantly lower in the control than in the experimental arm among patients with BM, with HR of 0.51 in these patients versus 0.87 overall.
Awada (2016) [18]	First	479	18	HER2-positive	Direct efficacy on BM not reported. Comparison between overall population and subgroup not reported.
Urruticoechea (2017) [19]	Second and subsequent	452	53	HER2-positive	Direct efficacy in BM not reported. PFS was significantly different between control and experimental arms among patients with BM, with a HR in these patients (0.29) that was lower than the overall HR (0.82).
Schmid (2018) [22]	First	902	61	Triple-negative	Direct efficacy in BM not reported. PFS was non-significantly different between control and experimental arms among patients with BM, with a HR in these patients (0.86) that was higher than the overall HR (0.81).
Murthy (2020) [23] and Lin (2020) [24]	Second and subsequent	612	219	HER2-positive	Direct efficacy in BM reported as central nervous system PFS, which was 40.2% in the experimental and 0% in the control arm at 1 year. PFS was significantly different between control and experimental arms among patients with BM, with a HR in these patients (0.48) that was lower than the overall HR (0.54). Overall survival was also significantly different between control and experimental arms among patients with BM, with a HR in these patients (0.58) that was lower than the overall HR (0.66).
Saura (2020) [25]	Third and subsequent	621	101	HER2-positive	Direct efficacy in BM not reported. The cumulative incidence of intervention for BM was significantly lower in the experimental than in the control arm.
Diéras (2020) [26]	First and subsequent	513	26	BRCA-mutated	Direct efficacy in BM not reported. PFS was not significantly different between control and experimental arms among patients with BM, but the HR in these patients (2.08) was higher than the overall HR (0.71).

* Liver/visceral and/or brain metastases. BM: brain metastases; BRCA: breast cancer gene; HR, hazard ratio; PFS, progression-free survival.

## Supplementary Materials

The following are available online at https://www.mdpi.com/article/10.3390/cancers13215306/s1, Table S1: The list of analyzed studies.
