# Supplementary material for: Patient Eligibility and Results for Brain Metastasis in Phase 3 Trials of Advanced Breast Cancer: A Scoping Review
Source: Cancers (Basel). 2021 Oct 22;13(21):5306. doi: 10.3390/cancers13215306 (PMC8582366; doi:10.3390/cancers13215306)
Supplement: Supplementary file 1 [file cancers-13-05306-s001.zip › cancers-1376411-supplementary.pdf]

Supplementary Table S1. The list of analysed studies.

No.	PMID	Journal	Year	Tumour phenotype	Therapy	N arms	N patients	BM allowance
1.	9469328	J Clin Oncol	1998	4	2	3	551	4
2.	9649142	Br J Cancer	1998	9	1	2	259	5
3.	9652764	Br J Cancer	1998	4	1	2	258	2
4.	9681078	Ann Oncol	1998	4	2	3	555	4
5.	9850014	J Clin Oncol	1998	4	1	2	303	4
6.	10091793	Cancer	1999	4	1	2	177	4
7.	10211086	Eur J Cancer	1999	9	3	2	415	4
8.	10334524	J Clin Oncol	1999	4	1	2	365	2
9.	10334526	J Clin Oncol	1999	9	1	2	392	4
10.	10458218	J Clin Oncol	1999	1	2	2	452	4
11.	10458219	J Clin Oncol	1999	4	2	3	368	4
12.	10550134	J Clin Oncol	1999	9	1	2	563	2
13.	10561296	J Clin Oncol	1999	9	1	2	326	4
14.	10561297	J Clin Oncol	1999	4	1	2	209	2
15.	10615229	Eur J Cancer	1999	4	1	2	282	4
16.	10637238	J Clin Oncol	2000	1	3	2	231	5
17.	10673513	J Clin Oncol	2000	4	1	2	331	5
18.	10674879	Breast Cancer Res Treat	1999	4	1	2	151	4
19.	10735887	J Clin Oncol	2000	1	2	2	769	4
20.	10856098	J Clin Oncol	2000	4	1	2	303	4
21.	10942095	Breast Cancer Res Treat	2000	4	2	3	313	2
22.	10963640	J Clin Oncol	2000	9	1	3	417	5
23.	11078487	J Clin Oncol	2000	1	2	2	668	4
24.	11078488	J Clin Oncol	2000	1	2	2	353	4
25.	11181656	J Clin Oncol	2001	4	1	2	460	4
26.	11230490	J Clin Oncol	2001	4	1	2	297	4
27.	11248153	New Engl J Med	2001	2	4	2	469	2
28.	11251000	J Clin Oncol	2001	9	1	2	267	2
29.	11261827	Breast Cancer Res Treat	2001	1	2	2	217	4
30.	11304774	J Clin Oncol	2001	4	1	2	151	2
31.	11352951	J Clin Oncol	2001	1	2	2	907	4
32.	11378344	Eur J Cancer	2001	9	1	2	280	4
33.	11454883	J Clin Oncol	2001	4	2	3	602	4
34.	11815957	Cancer	2002	4	1	2	224	4
35.	12065558	J Clin Oncol	2002	4	1	2	511	4
36.	12088118	Breast Cancer Res Treat	2002	1	2	2	1354	4
37.	12118025	J Clin Oncol	2002	4	1	2	275	2
38.	12177098	J Clin Oncol	2002	4	2	2	400	4
39.	12177099	J Clin Oncol	2002	1	2	2	451	4
40.	12419743	Ann Oncol	2002	4	1	1	260	4
41.	12439707	Br J Cancer	2002	9	1	2	176	4
42.	12586793	J Clin Oncol	2003	4	1	3	739	5
43.	12637459	J Clin Oncol	2003	9	1	2	429	4
44.	12721259	J Clin Oncol	2003	4	1	2	242	5
45.	12796608	Am J Clin Oncol	2003	1	2	2	238	4
46.	12797397	J Chemother	2003	4	1	3	131	4
47.	14556923	Eur J Cancer	2003	4	2	2	713	4
48.	14722035	J Clin Oncol	2004	4	2	2	305	4
49.	14998846	Ann Oncol	2004	4	1	2	509	2
50.	15117982	J Clin Oncol	2004	4	2	2	587	4
51.	15169793	J Clin Oncol	2004	9	1	3	474	2
52.	15197192	J Clin Oncol	2004	4	1	2	387	4

53.	15226326	J Clin Oncol	2004	4	1	2	144	4
54.	15305399	Cancer	2004	4	1	2	202	4
55.	15367413	Ann Oncol	2004	4	1	2	327	2
56.	15367414	Ann Oncol	2004	4	1	2	160	4
57.	15459210	J Clin Oncol	2004	4	1	2	301	5
58.	15570070	J Clin Oncol	2004	4	7	2	189	5
59.	15681523	J Clin Oncol	2005	4	5	2	462	4
60.	15726120	Br J Cancer	2005	4	1	2	201	2
61.	15735116	J Clin Oncol	2005	4	1	2	259	2
62.	15821120	Ann Oncol	2005	4	1	2	397	4
63.	16096436	Anticancer Drugs	2005	4	1	2	364	5
64.	16110015	J Clin Oncol	2005	4	1	2	449	5
65.	16172456	J Clin Oncol	2005	9	1	2	454	4
66.	16192591	J Clin Oncol	2005	4	1	2	216	4
67.	16293863	J Clin Oncol	2005	9	1	2	705	4
68.	16633363	Bone Marrow Transplant	2006	4	1	2	85	4
69.	16782917	J Clin Oncol	2006	2	4	2	196	2
70.	16921042	J Clin Oncol	2006	4	1	2	255	5
71.	17033039	J Clin Oncol	2006	4	1	3	527	4
72.	17045796	Eur J Cancer	2006	1	2	3	276	4
73.	17179098	J Clin Oncol	2007	5	1	2	227	5
74.	17192538	New Engl J Med	2006	2	4	2	324	2
75.	17329192	Lancet Oncol	2007	4	1	2	252	4
76.	17414459	Am J Clin Oncol	2007	4	1	2	341	4
77.	17611792	Breast Cancer Res Treat	2007	4	1	2	210	4
78.	17968020	J Clin Oncol	2007	4	1	2	752	4
79.	17971594	J Clin Oncol	2007	1	7	2	865	4
80.	17971595	J Clin Oncol	2007	1	2	2	352	4
81.	18037940	Bone Marrow Transplant	2008	4	1	2	308	4
82.	18160686	New Engl J Med	2007	4	5	2	722	4
83.	18483853	Breast Cancer Res Treat	2009	4	1	3	416	2
84.	19020973	Breast Cancer Res Treat	2009	1	9	2	157	5
85.	19254942	Ann Oncol	2009	4	1	3	441	2
86.	19273714	J Clin Oncol	2009	4	1	2	305	4
87.	19289619	J Clin Oncol	2009	2	4	2	156	5
88.	19687336	J Clin Oncol	2009	4	1	2	751	5
89.	19690954	Breast Cancer Res Treat	2010	2	4	2	112	5
90.	19768533	Breast Cancer Res Treat	2010	4	1	2	100	4
91.	19786658	J Clin Oncol	2009	2	4	2	1286	5
92.	19786670	J Clin Oncol	2009	2	4	2	207	4
93.	19819914	Ann Oncol	2010	4	1	2	237	4
94.	19906761	Ann Oncol	2010	4	1	2	286	2
95.	20089562	Ann Oncol	2010	4	1	2	236	4
96.	20124187	J Clin Oncol	2010	2	4	2	296	2
97.	20339913	Breast Cancer Res Treat	2010	4	9	2	482	4
98.	20361253	Breast Cancer Res Treat	2010	4	1	2	288	2
99.	20498403	J Clin Oncol	2010	4	5	3	736	4
100.	20530276	J Clin Oncol	2010	4	1	2	1221	5
101.	20855825	J Clin Oncol	2010	1	2	2	736	4
102.	20938664	Cancer Chemother Phar-macol	2011	1	2	2	234	5
103.	21084429	Ann Oncol	2011	9	1	2	475	4
104.	21115860	J Clin Oncol	2011	2	4	2	263	4
105.	21149659	J Clin Oncol	2011	2	4	2	284	4
106.	21251813	Eur J Cancer	2011	9	1	2	162	5
107.	21324184	BMC Cancer	2011	9	1	2	139	2

108.	21358207	Oncology	2010	4	1	2	102	2
109.	21358208	Oncology	2010	4	1	2	85	4
110.	21376385	Lancet Oncol	2011	4	1	2	762	2
111.	21383283	J Clin Oncol	2011	4	1	2	1237	4
112.	21569994	Clin Breast Cancer	2011	4	7	2	485	4
113.	21572124	Oncologist	2011	4	7	2	1028	5
114.	21937705	Ann Oncol	2012	4	1	2	148	2
115.	21990397	J Clin Oncol	2011	4	5	2	684	2
116.	22025143	J Clin Oncol	2011	4	1	3	323	1
117.	22084374	J Clin Oncol	2011	4	1	2	337	4
118.	22094937	Breast Cancer Res Treat	2012	9	1	2	287	4
119.	22149875	New Engl J Med.	2012	2	4	2	808	4
120.	22149876	New Engl J Med	2012	1	7	2	724	4
121.	22331954	J Clin Oncol	2012	4	9	2	593	4
122.	22370325	J Clin Oncol	2012	1	2	2	514	5
123.	22853014	New Engl J Med	2012	1	2	2	694	4
124.	23020162	New Engl J Med.	2012	2	4	2	991	2
125.	23233719	J Clin Oncol	2013	1	7	2	1112	5
126.	23312888	Lancet Oncol	2013	4	5	2	564	5
127.	23509322	J Clin Oncol	2013	2	4	2	444	4
128.	23537313	BMC Cancer	2013	4	1	4	241	2
129.	23569309	J Clin Oncol	2013	4	1	2	231	4
130.	23569311	J Clin Oncol	2013	2	5	2	424	4
131.	23715630	Breast Cancer Res Treat	2013	1	2	2	298	4
132.	23771714	Breast Cancer Res Treat	2013	4	1	2	340	2
133.	23857972	J Clin Oncol	2013	4	9	2	442	4
134.	23902874	Lancet Oncol	2013	1	2	2	723	5
135.	24401928	Ann Oncol	2014	2	1	2	363	2
136.	24504445	Ann Oncol	2014	4	1	2	78	5
137.	24742739	Lancet Oncol	2014	2	7	2	569	2
138.	24793816	Lancet Oncol	2014	2	4	2	602	2
139.	24888818	J Clin Oncol	2014	4	1	2	123	2
140.	25176223	Int J Oncol	2014	4	1	2	233	2
141.	25185099	J Clin Oncol	2015	4	9	2	1144	4
142.	25273342	Lancet Oncol	2014	4	5	2	494	2
143.	25273343	Lancet Oncol	2014	4	5	2	360	4
144.	25348000	J Clin Oncol	2014	1	4	2	324	5
145.	25349301	J Clin Oncol	2014	3	6	2	519	2
146.	25519041	Breast Cancer Res Treat	2015	4	5	2	227	2
147.	25605838	J Clin Oncol	2015	2	4	2	540	4
148.	25605862	J Clin Oncol	2015	4	1	2	1102	5
149.	25691671	J Clin Oncol	2015	1	5	2	374	4
150.	25779558	J Clin Oncol	2015	2	4	2	652	4
151.	25795409	Lancet Oncol	2015	3	1	2	240	3
152.	26030518	New Engl J Med.	2015	1	2	2	521	2
153.	26056183	J Clin Oncol	2015	4	1	3	900	2
154.	26092818	Lancet Oncol	2015	2	4	2	719	5
155.	26350351	Breast Cancer	2016	1	2	2	222	5
156.	26482278	Lancet Oncol	2015	4	1	2	840	2
157.	26617202	Lancet Oncol	2016	4	1	2	618	2
158.	26822398	Lancet Oncol	2016	2	4	2	508	2
159.	26916095	Ann Oncol	2016	1	5	2	117	2
160.	26927446	Breast Cancer Res Treat	2016	4	5	2	600	4
161.	26941199	Breast Cancer Res Treat	2016	9	1	3	180	2
162.	27078022	JAMA Oncology	2016	2	4	2	479	2
163.	27138575	J Clin Oncol	2016	4	5	2	350	5

164.	27359058	Oncotarget	2016	1	2	2	221	5
165.	27618821	Cancer Res Treat	2017	4	1	2	212	4
166.	27717303	New Engl J Med	2016	1	2	2	668	4
167.	27724870	BMC Cancer	2016	4	5	2	147	4
168.	27798749	Breast Cancer Res Treat	2017	4	1	2	210	2
169.	27803006	Ann Oncol	2017	4	9	2	416	5
170.	27817944	Eur J Cancer	2016	4	5	2	481	2
171.	27908454	Lancet	2016	1	2	2	462	2
172.	27918780	JAMA	2017	2	4	2	458	2
173.	27959613	New Engl J Med	2016	1	2	2	666	5
174.	27998961	Ann Oncol	2017	2	4	2	175	4
175.	28005247	Breast Cancer Res Treat	2017	4	1	2	162	5
176.	28056202	J Clin Oncol	2017	2	4	3	1095	5
177.	28209298	Lancet Oncol	2017	4	1	2	405	2
178.	28437161	J Clin Oncol	2017	2	4	2	452	2
179.	28576675	Lancet Oncol	2017	1	9	2	1147	2
180.	28578601	New Engl J Med	2017	4	6	2	302	5
181.	28580882	J Clin Oncol	2017	1	2	2	669	4
182.	28968163	J Clin Oncol	2017	1	2	2	493	4
183.	28830796	Clin Breat Cancer	2017	4	9	2	537	2
184.	29447329	Ann Oncol	2018	4	1	2	770	4
185.	29481630	Ann Oncol	2018	4	1	2	594	2
186.	29713086	Nat Med	2018	3	1	2	376	2
187.	29804902	Lancet Oncol	2018	1	8	2	672	4
188.	29860922	J Clin Oncol	2018	1	8	2	726	3
189.	29878040	Ann Oncol	2018	3	1	3	191	5
190.	30110579	N Engl J Med	2018	4	6	2	431	2
191.	30121808	Breast Cancer Res Treat	2018	4	1	2	420	2
192.	30345906	N Engl J Med	2018	3	9	2	902	2
193.	30568294	Br J Cancer	2018	2	4	2	707	2
194.	29223745	Lancet Oncol	2018	1	9	2	432	3
195.	30745582	Br J Cancer	2019	4	1	2	436	5
196.	30928806	Eur J Cancer	2019	4	1	2	530	5
197.	31028610	Breast Cancer Res Treat	2019	4	1	2	235	3
198.	31036468	Lancet Oncol	2019	1	9	2	365	2
199.	31091374	N Engl J Med.	2019	1	9	2	572	2
200.	31825569	N Engl J Med	2019	2	4	2	612	2
201.	29458237	Cancer Res Treat	2019	4	1	2	221	2
202.	31841354	J Clin Oncol	2019	3	9	2	140	2
203.	32564260	Breast Cancer Res Treat	2020	2	8	2	243	5
204.	32578279	Eur J Cancer Care	2020	2	4	2	166	2
205.	32678716	J Clin Oncol	2020	2	4	2	621	2
206.	32819305	BMC Cancer	2020	2	4	2	225	2
207.	32822287	J Clin Oncol	2020	2	4	3	355	5
208.	32861273	Lancet Oncol	2020	4	6	2	513	2
209.	33186740	Ann Oncol	2020	1	9	2	516	5
210.	33278935	Lancet	2020	3	9	2	847	2

Phenotype: 1, hormone-receptor-positive alone; 2, HER-2-positive alone; 3, triple-negative alone; 4, mixed; 9; unknown; Therapy: 1, chemotherapy; 2, endocrine therapy; 3, chemotherapy + endocrine therapy; 4, anti-HER therapy; 5, bevacizumab; 6, PARP inhibitors; 7, mTOR inhibitors; 8, CDK 4/6 inhibitors; 9, others; BM allowance: 1, allowed, no restrictions; 2, allowed, with restrictions; 3, allowed, restrictions not specified; 4, not allowed; 5, allowance not reported.
